# Involvement of *MID1-COMPLEMENTING ACTIVITY 1* encoding a mechanosensitive ion channel in prehaustorium development of the stem parasitic plant *Cuscuta campestris*

**DOI:** 10.1093/pcp/pcaf009

**Published:** 2025-01-17

**Authors:** Jihwan Park, Kyo Morinaga, Yuma Houki, Ayako Tsushima, Koh Aoki

**Affiliations:** Graduate School of Agriculture, Osaka Metropolitan University, 1-1 Gakuen-Cho, Naka-Ku, Sakai, Osaka 599-8531, Japan; Graduate School of Agriculture, Osaka Metropolitan University, 1-1 Gakuen-Cho, Naka-Ku, Sakai, Osaka 599-8531, Japan; College of Life and Environmental Sciences, Osaka Prefecture University, 1-1 Gakuen-Cho, Naka-Ku, Sakai, Osaka 599-8531, Japan; Graduate School of Agriculture, Osaka Metropolitan University, 1-1 Gakuen-Cho, Naka-Ku, Sakai, Osaka 599-8531, Japan; Graduate School of Agriculture, Osaka Metropolitan University, 1-1 Gakuen-Cho, Naka-Ku, Sakai, Osaka 599-8531, Japan

**Keywords:** *Cuscuta campestris*, host-induced gene silencing, *MCA1*, mechanosensitive ion channel, parasitic plants, prehaustorium

## Abstract

**Parasitic plants pose a substantial threat to agriculture as they attack economically important crops. The stem parasitic plant *Cuscuta campestris* invades the host’s stem with a specialized organ referred to as the haustorium, which absorbs nutrients and water from the host. Initiation of the parasitic process in *C. campestris* requires mechanical stimuli to its stem. However, the mechanisms by which *C. campestris* perceives mechanical stimuli are largely unknown. Previous studies have shown that mechanosensitive ion channels (MSCs) are involved in the perception of mechanical stimuli. To examine if MSCs are involved in prehaustorium development upon tactile stimuli, we treated *C. campestris* plants with an MSC inhibitor, GsMTx-4, which resulted in a reduced density of prehaustoria. To identify the specific *MSC* gene involved in prehaustorium development, we analyzed the known functions and expression patterns of *Arabidopsis MSC* genes and selected *MID1-COMPLEMENTING ACTIVITY 1* (*MCA1*) as a primary candidate. The MSC activity of *CcMCA1* was confirmed by its ability to complement the phenotype of a yeast *mid1* mutant. To evaluate the effect of *CcMCA1* silencing on prehaustorium development, we performed host-induced gene silencing using *Nicotiana tabacum* plants that express an artificial microRNA-targeting *CcMCA1*. In the *CcMCA1*-silenced *C. campestris*, the number of prehaustoria per millimeter of stem length decreased, and the interval length between prehaustoria increased. Additionally, the expression levels of known genes involved in prehaustorium development, such as *CcLBD25*, decreased significantly in the *CcMCA1*-silenced plants. The results suggest that *CcMCA1* is involved in prehaustorium development in *C. campestris***.

## Introduction

Parasitic plants cause considerable damage to agriculture by attacking economically important crop plants (Parker 2013). Given the extensive damage caused by the stem parasite *Cuscuta campestris* to a broad range of host crops, studies on the parasitic mechanisms of this species have actively been conducted (Lanini and Kogan 2005).

*Cuscuta campestris* forms a specialized organ, or haustorium, to obtain water and nutrients from host plants (Kuijt 1977). After *C. campestris* attaches to and coils around the host stem, the development of the haustorium primordium, or prehaustorium, begins (Lee 2007). This initial process is triggered by light and mechanical stimuli (Tada et al. 1996). In seedlings, coiling and haustorium development are promoted by blue and far-red light and inhibited by red light; however, in mature *C. campestris* stems, stem development is not completely inhibited by red light (Yokoyama et al. 2023). Thus, light is a regulatory factor affecting the onset of parasitic development in a developmental stage-dependent manner.

In addition to light, mechanical stimuli have also been demonstrated to induce prehaustorium development in *C. campestris* (Tada et al. 1996). When the stem is subjected to physical pressure, e.g. from a stack of plastic or glass plates, prehaustoria develops without contact with living host plants (Olsen et al. 2016, Kaga et al. 2020). In the congeneric *C. japonica*, haustoria are projected toward solid vertical objects, such as acrylic rods or cotton swabs, responding to mechanical stimuli that arise as the plant coils around these structures (Tada et al. 1996, Bernal-Galeano et al. 2022). Organogenesis in response to mechanical stimuli has been reported in nonparasitic plants, such as the formation of lateral roots (LRs) and adventitious roots. In *Arabidopsis thaliana*, bending forces on roots induce a transient increase in Ca^2+^ concentration within the pericycle, which subsequently leads to the development of LRs (Ditengou et al. 2008, Richter et al. 2009). Similarly, in *Brachypodium distachyon*, wind-induced lodging causes leaf nodes to contact the ground, which in turn stimulates the development of adventitious roots (Nam et al. 2020).

Mechanosensitive ion channels (MSCs) play an important role in perceiving mechanical stimuli. Mechanical forces associated with coiling, bending, and touch generate tension on the plasma membrane, causing distortions that are transmitted through the lipid bilayer to ion channels. This distortion of the lipid bilayer alters the conformation of the ion channels, switching them between open and closed states (Hamilton et al. 2015). In *A. thaliana*, a double mutant lacking both *MSCS-LIKE2* (*MSL2*) and *MSL3* has been reported to show higher accumulation levels of the osmoprotectant proline, even under normal growing conditions (Wilson et al. 2014). Additionally, a gain-of-function mutant of *MSL10* is implicated in early signal transduction related to wound response and jasmonic acid synthesis (Zou et al. 2016). Furthermore, a double mutant of *A. thaliana MID1-COMPLEMENTING ACTIVITY 1* (*MCA1*) and *MCA2* exhibits growth retardation under high Mg^2+^ concentrations (Yamanaka et al. 2010). These findings underscore the significant roles *MSC*s play in the physiological and developmental regulation in *A. thaliana*. However, it remains unknown whether *MSC* genes are involved in initiating prehaustorium development in parasitic plants.

In this study, we identified an *MSC* gene that is involved in prehaustorium development in *C. campestris*. To determine whether MSC proteins are involved in prehaustorium formation, we first treated the *C. campestris* stem with an MSC protein inhibitor at the early stage of parasitism. Next, we used a host-induced gene silencing (HIGS) approach to suppress the expression of *C. campestris MID1-COMPLEMENTING ACTIVITY 1* (*CcMCA1*). Silencing of *CcMCA1* repressed prehaustorium development and downregulated the expression of genes involved in haustorium formation. The results suggest that *CcMCA1* is involved in the prehaustorium development in *C. campestris*.

## Results

### MSC inhibitor treatment impairs the initiation of prehaustorium development

**Figure 1.**
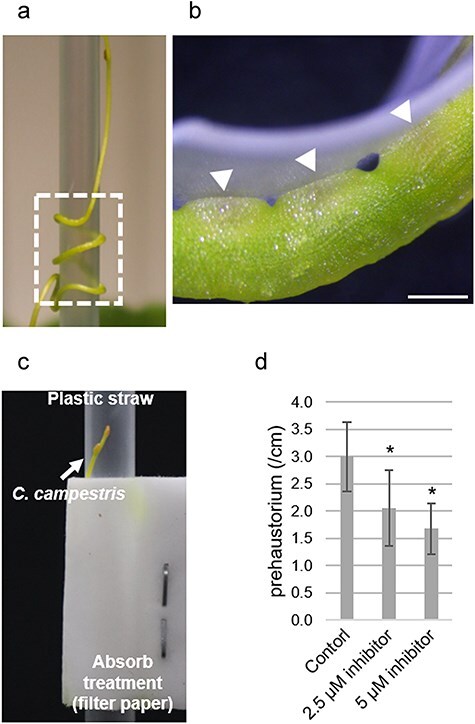
Impact of MSC inhibitor on prehaustorium development in *C. campestris*. (a) Setup of the artificial host system using a plastic straw. The rectangle indicates the area where filter paper is soaked with either water or the inhibitor solution. (b) Enlarged image of the attachment site. White arrowheads indicate prehaustoria. Scale bar = 1.0 mm. (c) Piece of filter paper saturated with the MSC inhibitor, GsMTx-4, at the site of attachment. (d) Comparison of prehaustorium numbers per centimeter of the stem at 48 haa. GsMTx-4-treated *C. campestris* developed fewer prehaustoria. The experiment was conducted using the following biological replicates: control (*n* = 8), 2.5 μM inhibitor (*n* = 7), and 5 μM inhibitor (*n* = 5). Data are represented as mean ± standard deviation. Asterisks indicate statistically significant differences estimated by Dunnett’s test (*P* < .05).

To investigate the involvement of MSC proteins in the development of prehaustoria induced by mechanical stimuli, we exposed *C. campestris* stems attached to a plastic straw serving as an artificial host to the MSC inhibitor, GsMTx-4, which blocks stretch-activated cation-selective channels (Gnanasambandam et al. 2017). The stems were secured to the straw using elastic surgical tape to apply consistent contact pressure (Fig. 1a and b). Treatment with 2.5 and 5 μM GsMTx4, concentrations within the effective range of the inhibitor, was administered (Hurst et al. 2009) (Fig. 1c). At 48 h after attachment (haa), the control stems treated with water developed an average of 3.00 ± 0.21 prehaustoria per centimeter of stem length. In contrast, treatment with 2.5 and 5 μM GsMTx4 significantly reduced the frequency of prehaustorium formation to 2.05 ± 0.21 and 1.68 ± 0.23, respectively (Fig. 1d). These results suggest that MSC protein activity is required for prehaustorium development in *C. campestris*.

### Prioritization of candidate *MSC* genes in *C. campestris*

Next, we focused on identifying the *C. campestris MSC* genes responsible for sensing mechanical stimuli to develop prehaustorium. According to a previous review, the *A. thaliana* genome encodes members of three MSC gene families: *MECHANOSENSITIVE CHANNEL OF SMALL CONDUCTANCE-LIKE* (*MSL*), *MCA1*, and *TWO-PORE POTASSIUM* (Hamilton et al. 2015). These *A. thaliana* genes were evaluated based on two criteria: (i) their loss of function affects their response to mechanical stimuli and (ii) their expression in the stem was confirmed. Four *A. thaliana* genes, *AtMSCS-LIKE2* (AT5G10490), *AtMSCS-LIKE3* (AT1G58200), *AtMSL10* (AT5G12080), and *AtMCA1* (AT4G35920) satisfied both of these criteria. In addition to these criteria, previous studies have reported that *At*MCA1 regulates hypocotyl growth in response to hypergravity, a form of mechanical stimulus (Hattori et al. 2020). We therefore selected *MCA1* as a primary candidate for further analysis. A Protein Basic Local Alignment Search Tool similarity search identified *CcMCA1* (Cc047049.t1, VFQ74305.1) as the closest homolog of *AtMCA1* in *C. campestris* (Supplementary Table S1).

### Suppression of prehaustorium development by silencing of *CcMCA1*

**Figure 2.**
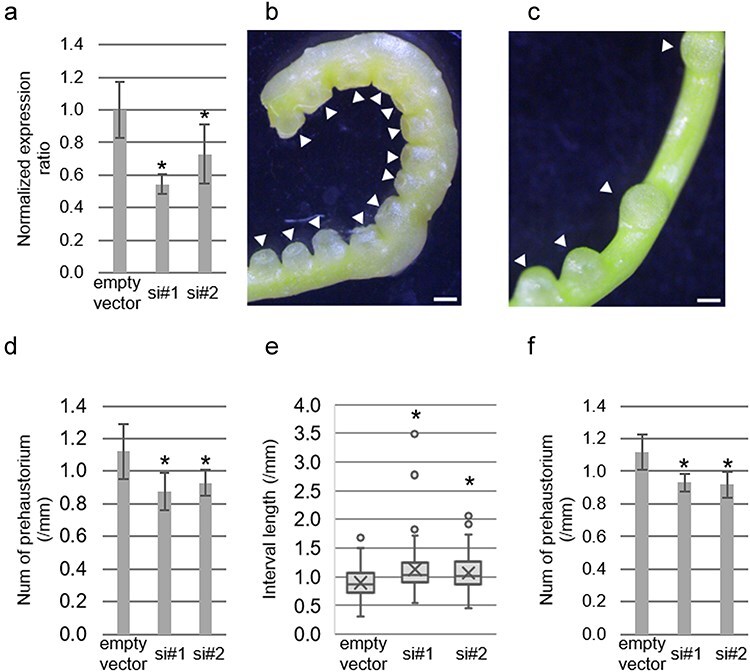
Inhibition of prehaustorium development by *CcMCA1*-silencing. (a) Relative expression level of *CcMCA1* in stems of *C. campestris* extended from two independent lines of T_2_  *N. tabacum* expressing amiRNA-targeting *CcMCA1*, compared with an *N. tabacum* line harboring an empty vector before attachment to wild-type *N. tabacum* as the secondary host. Expression levels were normalized against the average values of the vector control. Biological replicates were as follows: empty vector (*n* = 5), si#1 (*n* = 6), and si#2 (*n* = 5). (b and c) Morphological appearance of *C. campestris* stem at 72 haa to the wild-type *N. tabacum*. (b) Stem elongated from the *N. tabacum* line transformed with the empty vector. (c) Stem elongated from the *N. tabacum* line expressing amiRNA-targeting *CcMCA1* (si#2) at 72 haa. Arrowheads indicate prehaustoria. Scale bar = 1.0 mm. (d) The number of prehaustoria per centimeter of stem length of *C. campestris* attached to wild-type *N. tabacum* as the secondary host at 72 haa. The experiment was conducted using six biological replicates. (e) Interval between neighboring prehaustoria on stems of wild-type *N. tabacum* as the secondary host at 72 haa. Interval counts were as follows: empty vector (*n* = 162), si#1 (*n* = 123), and si#2 (*n* = 215). (f) Number of prehaustoria per centimeter of stem length in an *in vitro* haustorium induction system at 48 haa. Conditions included *CcMCA1*-silenced plants elongated from the amiRNA-expressing *N. tabacum* lines #1 (si#1) and #2 (si#2). Biological replicates were as follows: empty vector (*n* = 6), si#1 (*n* = 3), and si#2 (*n* = 5). Data are represented as mean ± standard deviation. Asterisks indicate statistically significant differences compared to the empty-vector control estimated by Dunnett’s test (*P* < .05).

To clarify whether *CcMCA1* is responsible for prehaustorium development, we knocked down *CcMCA1* using a HIGS method (Alakonya et al. 2012). Using RT-qPCR, we confirmed the expression of *CcMCA1* in the parasitizing stem of *C. campestris* (Supplementary Fig. S1). Consequently, *CcMCA1* transcripts were susceptible to the microRNA (miRNA)-based gene silencing. We used two host plants: the first one constitutively expresses artificial miRNA (amiRNA), and the second one was a wild-type plant, on which the effect of silencing was evaluated (Supplementary Fig. S2). Attaching to two independent lines of *Nicotiana tabacum*, engineered to express amiRNA-targeting *CcMCA1*, the expression level of *CcMCA1* was significantly reduced in the stems that elongated from the initial parasitizing sites compared to control plants that did not express amiRNA (Fig. 2a). This reduction in gene expression was associated with a noticeable phenotypic alteration in the development of prehaustoria. The *CcMCA1*-silenced plants exhibited a reduced number of prehaustoria per centimeter of stem length than the control plant (Fig. 2b–d, Supplementary Fig. S3). Additionally, the spacing between neighboring prehaustoria was longer in the *CcMCA1*-silenced plants than in the nonsilenced control plants (Fig. 2e). We investigated whether the reduced density of the prehaustorium was caused by delayed development resulting from the silencing of *CcMCA1*. We examined whether the prehaustorium could be initiated at a later time point, specifically at 120 haa, in the stems where *CcMCA1* was silenced. We could not find primordia of prehaustoria in the stem of long prehaustorial intervals, where no prehaustoria had developed at 72 haa (Supplementary Fig. S4). This result suggested that the prehaustorium development is not just delayed but repressed at the early stage of development in *CcMCA1*-silenced plants, even though we could not exclude the possibility that the prehaustorium development is delayed >120 h. Further analysis was conducted using an *in vitro* haustorium induction system, where stems were mechanically stressed by sandwiching between two glass plates. Under these conditions, the stems of the *CcMCA1*-silenced plants developed fewer prehaustoria compared to the controls at 48 haa (Fig. 2f). These findings underscore the critical role of *CcMCA1* in prehaustorium induction in *C. campestris*.

### *CcMCA1* rescues the mating pheromone-sensitive cell death phenotype in yeast *mid1* mutants

**Figure 3.**
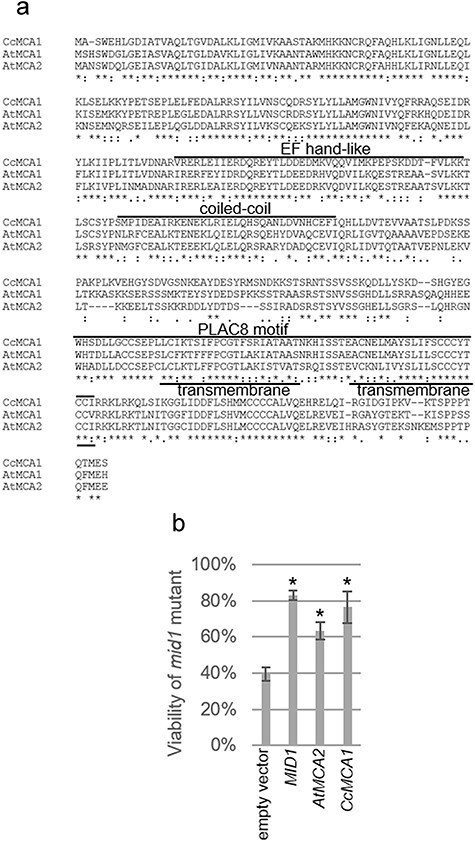
Amino acid sequence of *CcMCA1* and its complementation in yeast *mid1* mutant. (a) Amino acid sequence alignments of *CcMCA1*, *AtMCA1*, and *AtMCA2*. (b) Viability assessment of the yeast *mid1* mutant following transformation with the empty vector (control), yeast *MID1, AtMCA2*, and *CcMCA1*. The experiment included the following biological replicates: empty vector (*n* = 4), *MID1* (*n* = 4), *AtMCA2* (*n* = 3), and *CcMCA1* (*n* = 4). Data are represented as mean ± standard deviation. Asterisks indicate statistically significant differences estimated by Dunnett’s test (*P* < .05).

To determine whether *CcMCA1* functions as a homolog of *MCA1* genes, we compared the deduced amino acid sequence of *Cc*MCA1 with those of *A*tMCA1 (At4g35920) and *At*MCA2 (At2g17780) (Nakagawa et al. 2007). The *Cc*MCA1 sequence shared a high degree of identity with *At*MCA1 (78.75%) and *At*MCA2 (74.02%). Sequence alignment revealed that *Cc*MCA1, *AtMCA1*, and *AtMCA2* share conserved features, including an EF-hand-like motif, a calcium-binding domain, an ion transport domain, and a PLAC8 motif, all of which suggest that *Cc*MCA1 likely has mechanosensitive Ca^2+^ channel activity (Fig. 3a). To further assess the activity of *Cc*MCA1 as an MSC, we conducted a functional complementation assay using the yeast *Saccharomyces cerevisiae mid1* mutant. This mutant exhibits reduced viability in the presence of the mating pheromone, α-factor (Iida et al. 1994, Kanzaki et al. 1999). We transformed the *mid1* mutant with the yeast expression plasmid YEpTDHXho-*CcMCA1*, which harbors the *CcMCA1* cDNA driven by the yeast *TDH3* promoter. The results of this experiment indicated that *CcMCA1* effectively rescued the *mid1* mutant from the mating pheromone-induced cell death phenotype to the same extent as yeast *MID1* and *AtMCA2* (Fig. 3b). This outcome confirms that *CcMCA1* encodes a Ca^2+^ channel with the ability to complement the *mid1* mutation.

### Impact of *CcMCA1* silencing on the expression of haustorium development-related genes

**Figure 4.**
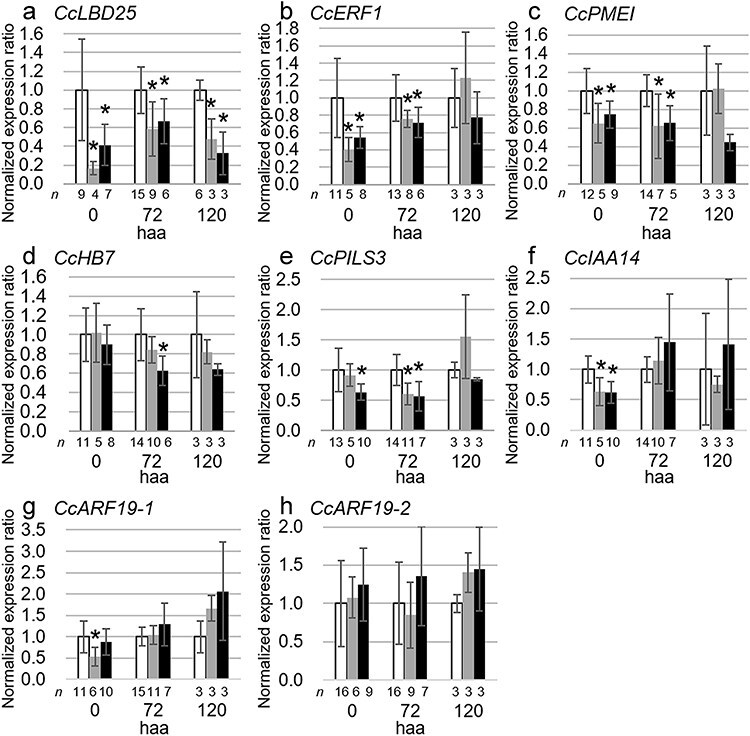
Impact of *CcMCA1* silencing on the expression of haustorium development-related genes. Relative expression levels of genes associated with haustorium development and auxin signaling in *C. campestris* stems. Expression was measured in stems attached to *N. tabacum* at 0, 72, and 120 haa. (a) *CcLBD25*, (b) *CcERF1*, (c) *CcPMEI*, (d) *CcHB7*, (e) *CcPILS3*, (f) *CcIAA14*, (g) *CcARF19-1*, and (h) *CcARF19-2*. Expression of genes was compared across three groups: *C. campestris* attached to the *N. tabacum* control line transformed with an empty vector (white), and those attached to the two independent lines of *N. tabacum* expressing amiRNA-targeting *CcMCA1* (line si#1 in gray and line si#2 in black). The vertical axis represents the relative expression level normalized to the reference gene, *CcRPS18*, and is further standardized to the mean values of the empty vector control. *n* represents the number of biological replicates. Data are represented as mean ± standard deviation. Asterisks indicate statistically significant differences as estimated by Dunnett’s test (*P* < .05).

Given that the *CcMCA1*-silenced plants developed fewer prehaustoria compared to the control plants, we investigated how *CcMCA1* silencing affects the expression of genes related to haustorium development (Jhu et al. 2021, 2022). We evaluated the expression levels of the genes at three time points: 0 haa, which was the time the *C. campestris* stem attached to the second host; 72 haa, when the prehaustoria grew and began to penetrate the second host; and 120 haa, when intrusive haustoria had developed in the second host (Shimizu and Aoki 2019; Supplementary Fig. S2). The analysis was conducted using stems elongated from two independent lines of *N. tabacum* expressing amiRNA-targeting *CcMCA1*, as well as from a control *N. tabacum* line. The expression level of the *C. campestris* homolog of *LATERAL ORGAN BOUNDARIES DOMAIN 25* (*CcLBD25*, Cc019141.t1, and VFQ91880.1), which has been suggested to regulate haustorium development through auxin signaling (Jhu et al. 2021), was significantly reduced in the *CcMCA1*-silenced plants at all three time points compared to the control plants (Fig. 4a). Next, we measured the expression levels of *C. campestris ETHYLENE RESPONSE FACTOR 1* (*CcERF1*, Cc002541.t1, and VFQ80528.1), *PECTIN METHYL-ESTERASE INHIBITOR* (*CcPMEI*, Cc038093.t1, and VFQ88734.1), and *HOMEOBOX 7* (*CcHB7*, Cc014209.t1, and VFQ99272.1), which are involved in haustorium penetration in the host cortex, and their silencing has been shown to halt this process (Jhu et al. 2022). The expression levels of *CcERF1* and *CcPMEI* were significantly reduced in *CcMCA1*-silenced plants at both 0 and 72 haa compared to the control plants, but no significant difference was observed at 120 haa (Fig. 4b and c). Conversely, *CcHB7* was downregulated only in the stems of *CcMCA1*-silenced plants from the amiRNA-producing *N. tabacum* line si#2 at 72 haa, showing no significant change in the *N. tabacum* line si#1 (Fig. 4d). These results show that *CcMCA1* silencing consistently reduced the expression level of *CcLBD25*, while *CcERF1* and *CcPMEI* decreased only in the preintrusive phase of prehaustorium development.

The downregulation of *CcLBD25* caused by *CcMCA1* silencing prompted us to investigate its impact on the expression levels of genes involved in auxin signaling in *C. campestris*. We first examined the expression level of *PIN-LIKES 3* (*CcPILS3*, Cc034373.t1, and VFQ99766.1), which encodes an auxin efflux carrier and coexpressed with *CcLBD25* (Jhu et al. 2022). The results showed a decreased expression level of *CcPILS3* in *CcMCA1*-silenced plants compared to the control plants only at 72 haa, but not at 0 or 120 haa (Fig. 4e). Subsequently, we evaluated the expression levels of the *C. campestris* homologs of *IAA14* and *ARF19*, which are transcriptional regulators known to regulate auxin-inducible expression of *LBD16* in *A. thaliana* (Okushima et al. 2007). Expression levels of *CcIAA14* (Cc038909.t1 and VFQ91216.1), *CcARF19-1* (Cc008555.t1 and VFQ92502.1), and *CcARF19-2* (Cc008622.t1 and VFQ92566.1) were also analyzed in the *CcMCA1*-silenced plants. No significant differences were observed compared to control plants at the three time points (Fig. 4f–h). These findings suggest that while the expression of *CcLBD25* is suppressed in *CcMCA1*-silenced plants, the transcriptional regulation of *CcIAA14* or *CcARF19* may not be associated with the suppression of *CcLBD25*.

## Discussion

### Role of *CcMCA1* in perceiving mechanical stimuli during haustorium development

Tada et al. (1996) described that the haustorium development of *C. japonica* can be initiated by contact with an acrylic rod under far-red light. However, far-red light alone did not induce haustorium development when *C. japonica* seedlings were incubated in test tubes without any supporting material to entwine around, demonstrating that mechanical stimuli are necessary for inducing haustorium development. Subsequent studies have consistently reaffirmed the necessity of mechanical stimuli for inducing haustoria in *C. campestris* (Bernal-Galeano et al. 2022). These findings have been supported by experiments using artificial host—or *in vitro* haustorium induction—systems (Olsen et al. 2016, Kaga et al. 2020, Jhu et al. 2021). However, the genes involved in perceiving mechanical stimuli have not been identified. In this study, we elucidated the involvement of MSC activity by employing an MSC inhibitor (Fig. 1d). We further identified *CcMCA1* as a gene critical for the mechanical induction of prehaustorium development (Fig. 2c–e). To the best of our knowledge, this is the first report to show that an *MSC* gene is implicated in the development of prehaustoria induced by mechanical stimuli.

Prehaustorium development in *C. campestris* is induced by two types of mechanical stimuli: coiling and clamping pressure. These induction processes may not entirely coincide, as evidenced by the differential effects of red light on haustorium development in mature *C. campestris* stems subjected to either coiling or clamping pressure (Yokoyama et al. 2023). Despite these variations, our results showed that the silencing of *CcMCA1* suppressed both coiling- and clamping-induced prehaustorium development (Fig. 2d–f), suggesting that *CcMCA1* is responsible for perceiving both types of mechanical stimuli. The involvement of *CcMCA1* likely pertains to its capacity to detect common changes, such as plasma membrane distortion, which aligns with previously proposed mechanisms (Hamilton et al. 2015).

The successful complementation of the mating pheromone-induced cell death phenotype in yeast *mid1* mutants strongly supports the function of *Cc*MCA1 as a mechanosensitive Ca^2+^ channel (Fig. 3b). Future measurements of Ca^2+^ uptake will provide further confirmation of the function of *Cc*MCA1. In *A. thaliana*, the constitutively expressed *At*MCA1 is localized in the plasma membrane, which is consistent with the presence of potential transmembrane segments in its amino acid sequence (Nakagawa et al. 2007). At the tissue level, *AtMCA1* is expressed in the stele and endodermis of the root (Yamanaka et al. 2010), suggesting that the *At*MCA1 protein perceives mechanical stimuli in these specific root cells. Although we confirmed that *CcMCA1* is expressed in the stem of *C. campestris* both before and after attachment to the host (Supplementary Fig. S1), the detailed spatial expression pattern of *CcMCA1* remains to be determined. Identifying the tissue-specific and subcellular localization of *Cc*MCA1 in *C. campestris* will be an important focus of future studies.

We note that there is another *MCA* homolog, Cc038057.t1 (VFQ88696.1), which has a high identity with *CcMCA1* in both protein (identity 99%) and nucleotide (identity 99%) sequence levels. Due to the high sequence identity, we have not successfully distinguished *CcMCA1* and Cc038057.t1 by RT-qPCR so far. Therefore, we could not exclude the possibility that Cc038057.t1 was silenced by the amiRNA-targeting *CcMCA1*, suggesting that Cc038057.t1 also contributes to the mechanical induction of prehaustorium development.

### Regulation of haustorium development-related genes downstream of *CcMCA1*

*CcLBD25* has been reported to play a pivotal role in haustorium development; *CcLBD25*-silenced plants exhibit a reduced number of prehaustoria in *C. campestris* (Jhu et al. 2021). LBD proteins, including *Cc*LBD25, are well-studied transcription factors regulated by the auxin signaling pathway (Okushima et al. 2007). In this study, we observed a significant reduction in the expression of *CcLBD25* in *CcMCA1*-silenced plants (Fig. 4a). This finding suggests a possible regulatory cascade where decreased prehaustorium density in *CcMCA1*-silenced plants is, at least partially, the consequence of *CcLBD25* repression.

*CcERF1* and *CcPMEI* have also been shown to regulate haustorium development, as silencing these genes leads to the cessation of haustorial penetration into the cortex of the host stem (Jhu et al. 2022). *ERF* functions as a secondary transcriptional regulator within the ethylene signaling pathway, activated by the primary transcriptional regulator *ETHYLENE INSENSITIVE 3*, which in turn induces the expression of various target genes involved in biotic stress responses (Solano et al. 1998). Interestingly, ethylene has been implicated in the emergence of adventitious roots in rice, where it promotes cell death in the epidermis overlaying the roots (Steffens et al. 2012). *PMEI* encodes an inhibitor of pectin methylesterase (PME). PME catalyzes the production of demethylesterified homogalacturonans, which are preferred substrates for calcium-dependent pectin-degrading enzymes (Sénéchal et al. 2015). Thus, *PMEI* and *PME* play critical roles in maintaining cell wall integrity by regulating the balance between pectin esterification and de-esterification (Daher and Braybrook 2015, Jobert et al. 2023). The observed reduction in expression levels of *CcERF1* and *CcPMEI* at 72 haa in *CcMCA1*-silenced plants (Fig. 4b and c) suggests that these genes may also play a role in prehaustorium development in response to mechanical stimuli. Given that silencing amiRNA was continuously supplied to the stem of *C. campestris*, the reduced repression of *CcERF1* and *CcPMEI* at 120 haa suggests that, while *CcMCA1* may be involved in regulating *CcERF1* and *CcPMEI* during preintrusive phase of the prehaustorium development, other factors are likely involved in the regulation of these genes.

Jhu et al. (2022) reported that the silencing of *CcHB7* leads to arrested haustorial penetration in the host stem. In *A. thaliana, AtHB7* is known to modulate abscisic acid (ABA) signaling by positively controlling the expression of the ABA receptor *PROTEIN PHOSPHATASE TYPE 2C*, which in turn negatively regulates ABA signaling (Valdés et al. 2012, Pehlivan 2019). In *CcMCA1*-silenced plants, the expression of *CcHB7* did not show consistent downregulation, with the exception at 72 haa in the *CcMCA1*-silenced plant line si#2 (Fig. 4d). Thus, it appears that *CcHB7* is not directly involved in the *CcMCA1*-dependent mechanical stimuli-induced prehaustorium development. However, the potential role of ABA signaling in this process cannot be completely ruled out.

### Signal transduction of mechanical stimuli during prehaustoria induction

Recent studies have suggested that haustorium development in parasitic plants may involve signal transduction mechanisms similar to those observed in LR development (Yoshida et al. 2019, Jhu et al. 2021). For example, during the initiation of LR development in *A. thaliana*, the PIN3 protein relocalizes to the inner plasma membrane of the endodermal cells, facilitating the efflux of auxin into the LR founder cells before the initiation of LR development (Marhavý et al. 2013). Subsequently, *LBD* transcription factors are specifically expressed in LR founder cells, where they regulate the initiation of LR development (Okushima et al. 2007). This regulation process includes the promotion of the expression of *PIN*s (Feng et al. 2012). In parasitic plants, *LBD* homologs have been reported to be upregulated at the parasitic interface of the root in parasitic plants such as *Thesium chinense* (Ichihashi et al. 2018) and *Striga hermonthica* (Yoshida et al. 2019). Our results showed that in *C. campestris*, the expression of *CcPILS3*, which encodes a putative auxin efflux carrier, along with *CcLBD25*, is reduced in *CcMCA1*-silenced plants only at 72 haa (Fig. 4e). This suggests a possible mechanistic pathway wherein the perception of mechanical stimuli by *CcMCA1* leads to a localized increase of auxin by *Cc*PILS3 in the early stage of prehaustorium development. We treated a parasitic site with 2,3,5-triiodobenzoic acid (TIBA) to test whether inhibiting polar auxin transport represses the prehaustorium development. However, the application of exogenous TIBA did not decrease the density of prehaustoria (Supplementary Fig. S5). To further understand the roles of polar auxin transport in the prehaustorium development, future studies should include the knock-down of *CcPILS3* and other auxin carriers.

Although our findings highlight a potential link between *CcMCA1* and auxin signaling, the specific pathway that activates *CcLBD25* remains unclear. In *A. thaliana*, LR development typically involves the activation of *LBD16* and *LBD29* through an auxin-dependent regulatory module comprising *IAA14* and *ARF7/19* (Okushima et al. 2007, Porco et al. 2016). In the present study, the expression of *CcIAA14* was not significantly downregulated at 72 haa by the silencing of *CcMCA1* (Fig. 4f). This observation corroborates findings in *A. thaliana*, where the expression of *IAA14* is less sensitive to indole-3-acetic acid (IAA) compared to other IAA-responsive genes (Abel et al. 1995). However, in this study, the expression levels of *CcARF19-1* and *CcARF19-2* did not differ significantly between the *CcMCA1*-silenced plants and the control plants (Fig. 4g and h). This observation suggests that, at least at the transcriptional level, the expression of *CcLBD25* may not be regulated by *CcIAA14* and *CcARF19*. Interestingly, in contrast to normal LR formation, mechanical stimuli-induced LR formation is also independent of the *IAA14-ARF7/19* module in *A. thaliana*, since LRs are still formed at bending sites in *arf7 arf19* double mutants (Ditengou et al. 2008), suggesting that *LBDs* can be activated even in the absence of *ARF7* and *ARF19*. Similarly, we speculate that the regulation of *CcLBD25* in the prehaustorium induction of *C. campestris* may also bypass the conventional *CcIAA14-CcARF7/19* regulatory module.

To search for potential regulatory factors of *CcLBD25*, we reanalyzed publicly available RNA-seq datasets for *C. campestris*, including accession numbers PRJDB9165 (Kaga et al. 2020) and PRJDB13193 (Yokoyama et al. 2022) (Supplementary Data S1). The resulting mapping data were then combined with the mapping data of PRJNA666991 (Bawin et al. 2022) and subjected to clustering analysis (Supplementary Fig. S6). For genes categorized into Clusters 1, 6, and 9, gene-by-gene Spearman’s rank correlation coefficients (*ρ*) were calculated. Notably, *CcLBD25* expression negatively correlated with *CcIAA14* (*ρ* = −0.62) and was not significantly correlated with *CcARF19-2* (*ρ* = −0.28) and *CcMCA1* (*ρ* = −0.30) (Supplementary Data S2 and S3). To further explore potential regulatory genes associated with *CcLBD25*, we analyzed the coexpression network of *C. campestris* genes (Supplementary Data S4). *CcLBD25* was found to cluster with a putative homolog of transcription factor genes such as *WRKY* family (VFQ61878.1, *ρ* = 0.81, *P* = 2.6e-17) and *AT-hook* motif nuclear-localized protein family (VFQ77022.1, *ρ* = 0.81, p= 1.8e-17) within coexpression module 21 (Supplementary Data S4 and S5, Supplementary Fig. S7). These results imply that mechanical stimuli-induced signals from *CcMCA1* might be transmitted to *CcLBD25* through alternative regulatory factors, although further studies are required to address this possibility.

**Figure 5.**
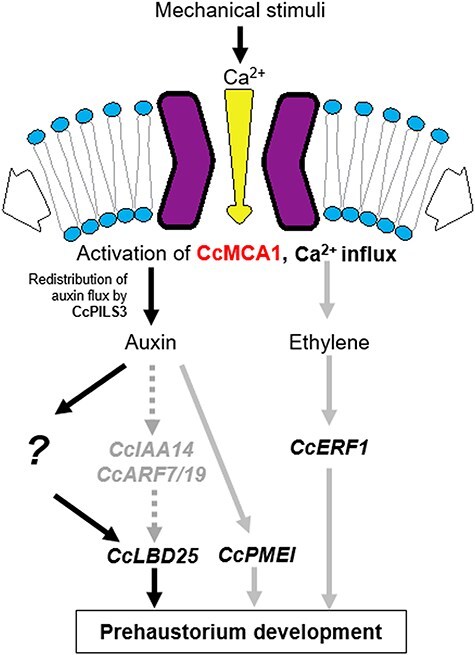
Hypothetical model of mechanical stimuli-induced prehaustorium development in *C. campestris*. The mechanical stimuli stretch the plasma membrane and activate *Cc*MCA1, leading to a Ca^2+^ influx into the cell. This influx is hypothesized to promote auxin accumulation in putative founder cells, mediated by the auxin efflux carrier *Cc*PILS3. Auxin is proposed to activate *CcLBD25* via an as yet unidentified signaling pathway (indicated by “?”), although the involvement of *CcIAA14* and *CcARF19* was not excluded. The upregulation of *CcLBD25* is believed to trigger prehaustorium development. *CcPMEI* and *CcERF1* may be regulated downstream of *CcMCA1*, although this regulation weakened in the intrusive phase of haustorium development. Gray arrows indicate the regulatory relation only in the early preintrusive phase of prehaustorium development. Gray-dotted arrows indicate that the *CcIAA14-CcARF19* regulatory module did not coexpress with *CcLBD25*, suggesting its lack of involvement in regulating *CcLBD25*.

Based on our findings and previously published data, we speculate how prehaustorium development is induced by mechanical stimuli (Fig. 5). Mechanical stimuli distort the plasma membrane, activating *Cc*MCA1, which then leads to an influx of Ca^2+^. This influx initiates signaling pathways involving auxin and ethylene. In the auxin signaling pathway, we hypothesize that *CcLBD25* is transcriptionally regulated downstream of *CcMCA1*. The *CcIAA14-CcARF19* regulatory module did not coexpress with *CcLBD25*, implying that auxin activates *CcLBD25* through an unidentified signaling pathway, although we cannot exclude the potential involvement of *CcIAA14* and *CcARF19. CcPMEI, CcERF1*, and *CcPILS3* are likely regulated downstream of *CcMCA1* during the early phase of prehaustorium development. However, this association seems to weaken as the prehaustorium begins to penetrate the host. This suggests that factors other than *CcMCA1* might be involved in regulating these genes. The detailed mechanisms linking *CcMCA1* to ethylene signaling remain unclear. Future research aimed at elucidating the downstream factors of *CcMCA1* will enhance our understanding of the mechanisms underlying the perception of mechanical stimuli and subsequent prehaustorium development in *C. campestris*.

## Materials and Methods

### Plant materials

*Cuscuta campestris* seeds were germinated and parasitized onto *N. tabacum* as described previously (Hozumi et al. 2017). Both plant species were cultivated at 25°C under a photoperiod consisting of 16 h of light and 8 h of dark. For experimental use, ∼5 cm-long excised stems of *C. campestris* containing an apical shoot meristem that had been propagated by being parasitized to *N. tabacum* were selected. To confirm whether *CcMCA1* was expressed in wild-type *C. campestris*, stems were harvested at 0, 12, 24, and 48 haa.

### GsMTx-4 inhibitor treatment

GsMTx-4 (Peptide Institute, Osaka, Japan) was dissolved in distilled and deionized water to make a 5-μM stock solution and kept at −20°C. The stems of *C. campestris* were attached to a plastic straw (polypropylene, diameter 10 mm) and wrapped with a piece of fiber filter paper (GA-100, 110 mm, ADVANTEC, Tokyo, Japan) soaked in either distilled water (control) or the inhibitor solution (experiment), illuminated with blue light for 1 h, and kept in darkness for 24 h, before being incubated at 22°C under a 16-/8-h light/dark cycle. The number of prehaustoria was counted at 48 haa using a stereomicroscope (SZX16, Olympus, Tokyo, Japan). More than five biological replicates were assessed for each experimental treatment (i.e. 2.5 and 5 μM) and the control treatment (i.e. distilled water).

### Prioritization of *MSC* genes

A list of *Arabidopsis MSC* genes was sourced from Hamilton et al. (2015). The electronic Fluorescent Pictograph browser (http://bar.utoronto.ca/efp/cgi-bin/efpWeb.cgi) (Winter et al. 2007) was used to investigate the expression of the *Arabidopsis MSC* genes in the stem. *Cuscuta campestris MSC* candidates were obtained by comparing all protein sequences from *C. campestris* (available in cucam_0.32.annot.protein.fasta.gz at http://www.plabipd.de/project_cuscuta2/start.ep) (Vogel et al. 2018) to a BLASTP search against *A. thaliana* proteins listed in Araport11_pep_20220914.gz (https://www.arabidopsis.org) (Huala et al. 2001) by *e*-value < 1.0e-50.

### RNA extraction and RT-qPCR

Total RNA was extracted from *C. campestris* stems, which were elongated from the initial parasitic site of *N. tabacum* stem, at 0, 72, and 120 haa to the second host plant of wild-type *N. tabacum* (Supplementary Fig. S2), using the RNeasy Plant Mini Kit (Qiagen, Hilden, Germany) and subsequently treated with a TURBO DNA-free^TM^ Kit (Thermo Fisher Scientific, Waltham, MA, USA) according to the manufacturer’s instructions. cDNA was synthesized using ReverTra Ace-α- (Toyobo, Osaka, Japan) and the supplied oligo(dT) primer.

RT-qPCR was performed using Fast SYBR^TM^ Green Master Mix (Thermo Fisher Scientific) and a StepOnePlus Real-time PCR System (Thermo Fisher Scientific). For plotting a standard curve of the targeted genes, cDNA was serially diluted by 1:5, 1:25, 1:125, and 1:625. Expression levels of the targeted genes were normalized to the *C. campestris RIBOSOMAL PROTEIN S18(CcRPS18)*. Primers used for RT-qPCR are shown in Supplementary Table S1.

### amiRNA constructs

An amiRNA sequence targeting *CcMCA1* was designed using the WMD3-Web MicroRNA Designer (http://wmd3.weigelworld.org/cgi-bin/webapp.cgi) following the guidelines provided on the WMD3-Web protocol pages (Schwab et al. 2006). The obtained amiRNA sequence (5ʹ-TTAGAGAAAACCTGCATGCTC-3ʹ) was synthesized and cloned into the pRS300 vector (Addgene, Watertown, MA, USA). The amiRNA fragment was then ligated into the binary vector pBI121, previously digested with SacI-HF (New England Biolabs, Ipswich, MA, USA) and SalI-HF (New England Biolabs) using Ligation High ver. 2 (Toyobo) to express the amiRNA precursor under the cauliflower mosaic virus (CaMV) 35S promoter. The constructed binary vector plasmid containing the amiRNA sequence was transformed into the *Agrobacterium* strain GV3101 for subsequent plant transformation. Primers used for constructing the vector are shown in Supplementary Table S1.

### Transformation of *N. tabacum* and HIGS

Agrobacterium strain GV3101 harboring the binary vector was cultured at 37°C by shaking at 180 rpm in liquid Luria-Bertani medium supplemented with 50 mg/l kanamycin, 50 mg/l gentamycin, and 25 mg/l rifampicin. The culture was grown until the optical density 600 nm (OD600) reached 1.0. For transformation, healthy leaves of *N. tabacum* grown at 25°C were cut into 1 cm squares. These leaf tissues were then inoculated with the *Agrobacterium* suspension and cultured on solid Murashige and Skoog (MS) medium containing 3% (w/v) sucrose, 1 mg/l 6-benzylaminopurine (BAP) (Nacalai Tesque, Kyoto, Japan), and 0.1 mg/l IAA (Nacalai Tesque). The inoculated tissues were incubated at room temperature in the dark for 2 days. Subsequently, the leaf tissues were transferred to fresh solid MS medium containing 3% (w/v) sucrose, 1 mg/l BAP, 0.1 mg/l IAA, 100 mg/l kanamycin (FUJIFILM Wako Pure Chemical Industries, Ltd, Osaka, Japan), and 12.5 mg/l meropenem (Sumitomo Dainippon Pharma, Osaka, Japan). These were cultured at 25°C under continuous light for ∼30 days to induce callus formation and shoot regeneration. The regenerated shoots, measuring 2 to 3 cm in height, were transferred to MS medium containing 3% (w/v) sucrose, 100 mg/l kanamycin, and 12.5 mg/l meropenem to promote root regeneration. Following successful root development, the transgenic plants were transplanted into soil (Sukoyaka-baido, Yanmar, Osaka, Japan) mixed with an equal volume of vermiculite (Nittai, Aichi, Japan) to facilitate further growth. To select for transgenic progeny, the T1 seeds were germinated on an MS medium containing 100 mg/l kanamycin. As described earlier, we also transformed *N. tabacum* with an *Agrobacterium* strain harboring an empty binary vector to serve as a control. To induce silencing of *CcMCA1* in *C. campestris*, a 5-cm excised stem section of *C. campestris* containing an apical shoot meristem that had been propagated on wild-type *N. tabacum* was parasitized onto the transgenic *N. tabacum* expressing the amiRNA-targeting *CcMCA1*. The stem of *C. campestris* was then elongated to ∼20 cm long for 1 week, and then the region 1–2 cm below the stem tip was attached to the stem of another 3-month-old wild-type *N. tabacum* (Supplementary Fig. S2), as described previously (Sultana et al. 2021).

### Measurement of prehaustorium density and interval length

To quantify prehaustorium density and interval length, stems of *C. campestris* parasitizing the transgenic *N. tabacum* lines were collected at 72 and 120 haa, and images were captured under a stereomicroscope (SZX 16, Olympus). For the *in vitro* haustorium induction system, stems elongated from each transgenic *N. tabacum* line were sandwiched between two cover glasses with two 0.4-mm-thick plastic spacers placed between them to maintain a fixed gap. Images of the stems were captured using the stereomicroscope at 48 haa. The interval length between prehaustoria was subsequently measured using ImageJ software (https://imagej.net/ij/) (Schneider et al. 2012).

### Yeast *mid1* complementation assay

Total RNA extraction and cDNA synthesis from *C. campestris* were performed, as previously described. The cDNA for *CcMCA1* was amplified using primers listed in Supplementary Table S2. The resulting amplified DNA fragment was purified using a Wizard^®^SV Gel and PCR Clean-Up System (Promega, Madison, WI, USA) and cloned into pCR^™^-Blunt II-TOPO^®^ vector using a Zero Blunt^®^ TOPO^®^ PCR Cloning Kit (Thermo Fisher Scientific). To facilitate subcloning, recognition sequences for BamHI and NotI were added at the 5ʹ and 3ʹ ends of the *CcMCA1* cDNA, respectively, by PCR using primers 5ʹ-GGATCCATGGCGTCGTGGG-3ʹ and 5ʹ-GCGGCCGCTTAGGATTCCAT-3ʹ, with BamHI and NotI sites denoted by underlines. Subsequent digestion of the resulting PCR products with BamHI (New England Biolabs) and NotI (New England Biolabs) facilitated their insertion into the YEplac181-based multicopy expression vector, YEpTDHXho (Gietz and Sugino 1988, Hashimoto et al. 2004), under the control of *TDH*3 promoter. The plasmid was designated as YEpTDHXho-*CcMCA1*.

The yeast strain H311 (*MAT*a *mid1*-Δ*5::HIS3 his3*-Δ*1 leu2-3,112 trp1-289 ura3-52 sst1-2*), characterized by its impaired Ca^2+^ influx (Tada et al. 2003), was used for transformation using the Yeastmaker^TM^ Yeast Transformation System 2 (Takara Bio, Shiga, Japan). As positive controls, YCpS-MID1 (*MAT*a *LEU2 CEN4 ARS1 MID1*p:*MID1 amp*^r^) (Iida et al. 2017) and YEpTDHXho-*MCA2* (Yamanaka et al. 2010) were used.

The yeast cells were cultivated in SD.Ca100 medium (Iida et al. 1990, 1994), which contains 100 μM CaCl_2_ as the Ca^2+^ source, at 30°C until they reached the exponentially growing phase (2 × 10^6^ cells/ml). Subsequently, the cells were incubated with 6 μM α-factor (Funakoshi, Tokyo, Japan) for 8 h to assess their viability. The viability was evaluated using the methylene blue staining method, as described previously (Iida et al. 1990).

### Reanalysis of RNA-seq data

RNA-seq data from BioProject nos PRJDB9165 (DRA009453, Kaga et al. 2020) and PRJDB13193 (Yokoyama et al. 2022) were preprocessed, as previously described (Yokoyama et al. 2022), and independently mapped to *C. campestris* transcriptome from the assembly GCA_900332095.2 (Vogel et al. 2018) using HISAT2 (Kim et al. 2015). These mapping results were then combined with data from “ppl13628-sup-0002-AppendixS1.xlsx” (PRJNA666991, Bawin et al. 2022) and renormalized. Gene expression patterns were clustered using the iDEP 2.01 Clustering tools (https://bioinformatics.sdstate.edu/idep/, Ge et al. 2018). Spearman’s rank correlation coefficients were calculated with R statistical software using “corrplot” package (v4.1.2; R Core Team 2021). Coexpression modules were generated, and network files were obtained using iDEP 2.01 Network tools (parameters: most variable genes to include = 3000, Soft Threshold = 5, Min. Module Size = 20, Ge et al. 2018). Network graphs were visualized using Cytoscape software (Shannon et al. 2003). A cutoff value of the edge weight was 0.24. Designation of *C. campestris* genes was obtained by performing BLASTP using *A. thaliana* protein sequence dataset in The Arabidopsis Information Resource (Berardini et al. 2015) as queries against *C. campestris* protein sequence dataset (GCA_900332095.2, Vogel et al. 2018).

### Statistical analysis

Dunnett’s test was performed using Prism 10 (GraphPad Software, Boston, MA, USA).

## Supplementary Material

pcaf009_Supp

## Data Availability

The data underlying this article are available in the article and in its online supplementary material.
