# Supplementary material for: Involvement of MID1-COMPLEMENTING ACTIVITY 1 encoding a mechanosensitive ion channel in prehaustorium development of the stem parasitic plant Cuscuta campestris
Source: Plant Cell Physiol. 2025 Jan 17;66(3):400–10. doi: 10.1093/pcp/pcaf009 (PMC11957263; doi:10.1093/pcp/pcaf009)
Supplement: pcaf009_Supp [file pcaf009_supp.zip › suppl_data/pcp-2024-e-00196-File007.pdf]

Supplementary Table S1

BLASTP analysis of *Cuscuta campestris* proteins against the *Arabidopsis thaliana* (At) MSC proteins described in Hamilton et al. (2015).

Gene name	At protein	BLASTP tophit	e-value	bit score	At mutant phenotype
Mechanosensitive channel of small conductance-like (MSL)					
MSL1	At4g00290	VFQ94676.1 (Cc011107.t1)	0	545	
MSL2	At5g10490	VFQ88088.1 (Cc006069.t1)	0	701	*msl2* mutant; abnormal leaf shape. *msl2 msl3* double mutants showed enlarged chloroplast, abnormal plastids, and multiple chloroplast division rings.
MSL3	At1g58200	VFQ95019.1 (Cc011396.t1)	1.11E-130	388	
MSL4	At1g53470	VFQ85051.1 (Cc036587.t1)	0	776	
MSL5	At3g14810	VFQ86448.1 (Cc037233.t1)	0	789	*msl4 msl5 msl6 msl9 msl10* mutants lack MS ion channel activity in root protoplast.
MSL6	At1g78610	VFQ61470.1 (Cc020496.t1)	0	823	
MSL9	At5g19520	VFQ84734.1 (Cc004406.t1)	0	605	*msl9* mutants lack MS ion channel activity in root protoplasts.
MSL10	At5g12080	VFQ60779.1 (Cc019814.t1)	0	639	*msl10* mutants lack MS ion channel activity in root protoplasts.
Mid1-complementing activity (MCA)					
MCA1	At4g35920	VFQ74305.1 (Cc047049.t1)	0	569	*mca1* mutants impaired root penetration and response to mechanical stress.
MCA2	At2g17780	VFQ88696.1 (Cc038057.t1)	3.49E-174	493	*mca2* mutants reduced Ca^{2+} uptake.
Two-pore potassium (TPK)					
TPK1	At5g55630	VFQ84437.1 (Cc036402.t1)	1.03E-138	399	tpk1 mutants do not have rapid K^+ currents in shoot cells, slower ABA
