# Supplementary material for: Involvement of MID1-COMPLEMENTING ACTIVITY 1 encoding a mechanosensitive ion channel in prehaustorium development of the stem parasitic plant Cuscuta campestris
Source: Plant Cell Physiol. 2025 Jan 17;66(3):400–10. doi: 10.1093/pcp/pcaf009 (PMC11957263; doi:10.1093/pcp/pcaf009)
Supplement: pcaf009_Supp [file pcaf009_supp.zip › suppl_data/pcp-2024-e-00196-File008.pdf]

Supplementary Table S2. Primers used in this study.

**Primer name**	**Sequence (5'→3')**	**Purpose**
*CcMCA1*_ami_I miR-s	GATTAGAGAAAACCTGCATGCTCTCTCTCTTTTGTATTCC	amiRNA construct
*CcMCA1*_ami_II miR-a	GAGAGCATGCAGGTTTTCTCTAATCAAAGAGAATCAATGA	amiRNA construct
*CcMCA1*_ami_III miR*s	GAGAACATGCAGGTTATCTCTATTCACAGGTCGTGATATG	amiRNA construct
*CcMCA1*_ami_IV miR*a	GATAGAGATAACCTGCATGTTCTCTACATATATATTCCT	amiRNA construct
ami_OligoA	CTGCAAGGCGATTAAGTTGGGTAAC	amiRNA construct
ami_OligoB	GCGGATAACAATTTCACACAGGAAACAG	amiRNA construct
*CcMCA1*_Forward	GGAGTGAAAATGGCGTCGTG	qRT-PCR
*CcMCA1*_Reverse	TGAGCCCTCCTGAACTGGTA	qRT-PCR
*CcARF19-1*_Forward	CTCTGTGCTGGTGATGCTGT	qRT-PCR
*CcARF19-1*_Reverse	GAAAGGGCGGGTTGTTGC	qRT-PCR
*CcARF19-2*_Forward	TAGCTGGGACATACATATCAATATAAACAT	qRT-PCR
*CcARF19-2*_Reverse	ACACATGTTCAAAAGTGGTCTAGAGA	qRT-PCR
*CcPILS3*_Forward	CCCGCAGTGACGCTTGTAAT	qRT-PCR
*CcPILS3*_Reverse	CCGCAAAGAGGGAGCAGAAT	qRT-PCR
*CcERF1*_Forward	AAGGGGAGGCACTACAGGG	qRT-PCR
*CcERF1*_Reverse	CCCACCGCCAACATCCAAG	qRT-PCR
*CcPMEI*_Forward	CCTGGGCAAGATAAGGGCAA	qRT-PCR
*CcPMEI*_Reverse	GCAAGTGTCTGGGTCGGTTA	qRT-PCR
*CcHB7*_Forward	GCATCATCGGAAACCCAGGAA	qRT-PCR
*CcHB7*_Reverse	TCCACCTTGCCCTTTTGTTCT	qRT-PCR
*CcIAA14*_Forward	CAGCGACAAGGGAGCAGTTC	qRT-PCR
*CcIAA14*_Reverse	CACCGACAAGCATCCAATCAC	qRT-PCR
*CcLBD25*_Forward	CCACAAACACTAGCAGGACA	qRT-PCR
*CcLBD25*_Reverse	TGTATCTTGTGGGCTTTCTGAG	qRT-PCR
*CcRPS18*_qPCR-Forward	GCAGTACAAAAGCCATGGAAAAGGC	qRT-PCR
*CcRPS18*_qPCR-Reverse	TCCAGCGTCTGTGATGAAATTGGC	qRT-PCR
