# Supplementary material for: Involvement of MID1-COMPLEMENTING ACTIVITY 1 encoding a mechanosensitive ion channel in prehaustorium development of the stem parasitic plant Cuscuta campestris
Source: Plant Cell Physiol. 2025 Jan 17;66(3):400–10. doi: 10.1093/pcp/pcaf009 (PMC11957263; doi:10.1093/pcp/pcaf009)
Supplement: pcaf009_Supp [file pcaf009_supp.zip › suppl_data/pcp-2024-e-00196-File009.pdf]

Park et al.
Supplementary Figure S1

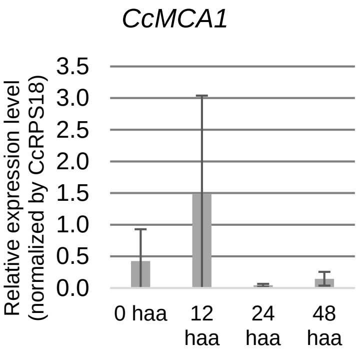

Supplementary Figure S1. Expression of *CcMCA1* after attaching to stems of wild type *Nicotiana tabacum.* haa; hours after attachment. Data are represented as means ± standard deviations of three replicates. There were not significant differences between mean values estimated by Dunnett's test ($p<0.05$).
