# Supplementary material for: Involvement of MID1-COMPLEMENTING ACTIVITY 1 encoding a mechanosensitive ion channel in prehaustorium development of the stem parasitic plant Cuscuta campestris
Source: Plant Cell Physiol. 2025 Jan 17;66(3):400–10. doi: 10.1093/pcp/pcaf009 (PMC11957263; doi:10.1093/pcp/pcaf009)
Supplement: pcaf009_Supp [file pcaf009_supp.zip › suppl_data/pcp-2024-e-00196-File010.pdf]

Park et al.
Supplementary Figure S2

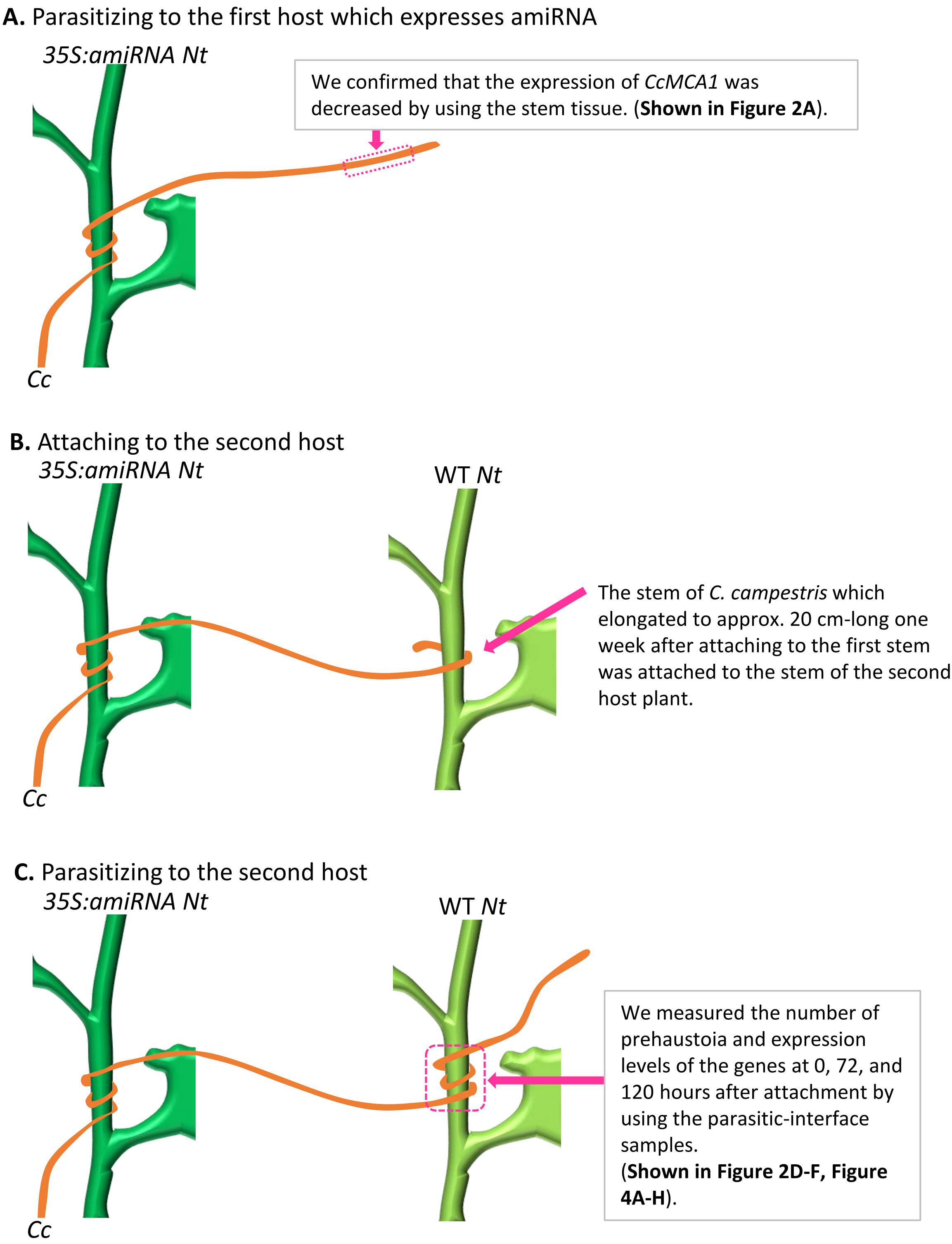

Supplementary Figure S2. Experimental setting of the host-induced gene silencing of *CcMCA1.* **(A)** *Cuscuta campestris* was parasitized to the first host, *Nicotiana tabacum* which expresses artificial microRNA (amiRNA) targeting *CcMCA1 under cauliflower mosaic virus 35S promoter* (*35S:amiRNA Nt*). Silencing effect on the *CcMCA1* expression was confirmed using stem tissue which extended from the attachment site (**Figure 2A**). **(B)** *C. campestris* stem, which stayed attached to the first host, was attached to the second host, wild type *N. tabacum* (WT *Nt*). **(C)** Parasitic interface tissues parasitized on WT *Nt* were harvested at 0, 72, and 120 hours after attachment, and the number of prehaustoria and expression levels of the haustorium development-related genes were measured (**Figure 2D-F, Figure 4A-H**).
