# Supplementary material for: Involvement of MID1-COMPLEMENTING ACTIVITY 1 encoding a mechanosensitive ion channel in prehaustorium development of the stem parasitic plant Cuscuta campestris
Source: Plant Cell Physiol. 2025 Jan 17;66(3):400–10. doi: 10.1093/pcp/pcaf009 (PMC11957263; doi:10.1093/pcp/pcaf009)
Supplement: pcaf009_Supp [file pcaf009_supp.zip › suppl_data/pcp-2024-e-00196-File011.pdf]

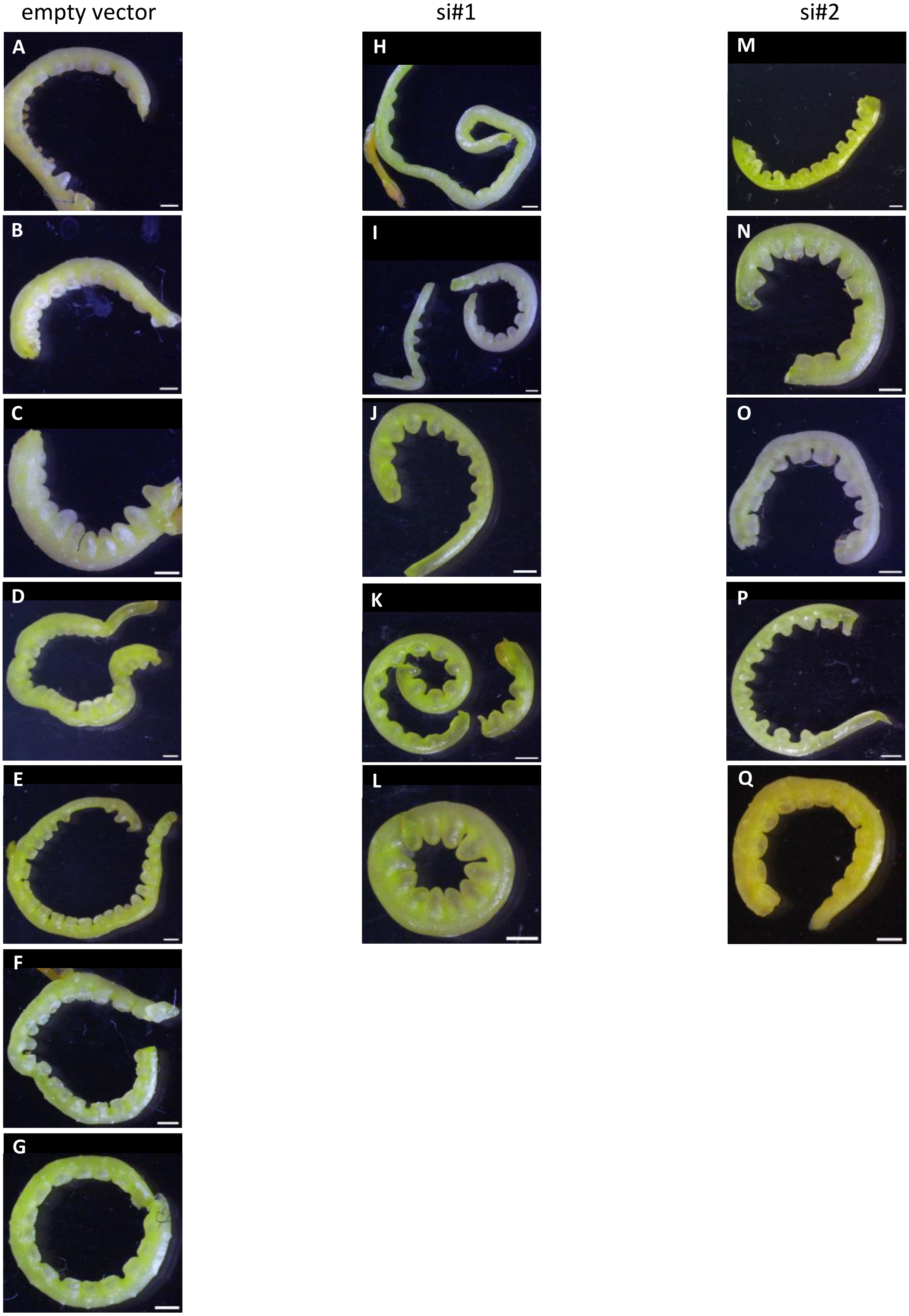

Supplementary Figure S3. Morphological appearance of *Cuscuta campestris* stem at 72 hours after attachment (haa) to the wild type *Nicotiana tabacum*. Stem elongated from the *N. tabacum* line transformed with the empty vector **(A-G)**, amiRNA targeting *CcMCA1* (si#1) **(H-L)**, amiRNA targeting *CcMCA1* (si#2) **(M-Q)**. Scare bar = 1.0 mm.
