# Supplementary material for: Involvement of MID1-COMPLEMENTING ACTIVITY 1 encoding a mechanosensitive ion channel in prehaustorium development of the stem parasitic plant Cuscuta campestris
Source: Plant Cell Physiol. 2025 Jan 17;66(3):400–10. doi: 10.1093/pcp/pcaf009 (PMC11957263; doi:10.1093/pcp/pcaf009)
Supplement: pcaf009_Supp [file pcaf009_supp.zip › suppl_data/pcp-2024-e-00196-File012.pdf]

Park et al.
Supplementary Figure S4

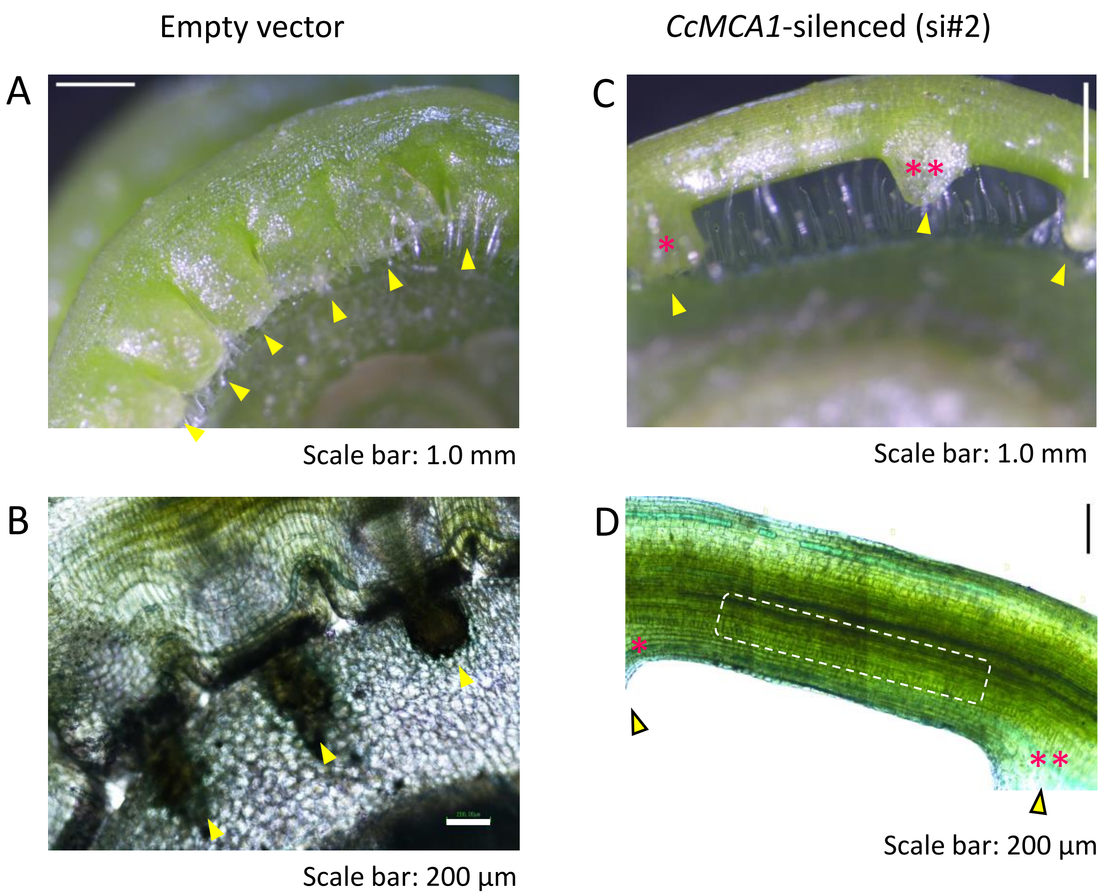

Supplementary Figure S4. Prehaustorium development in *CcMCA1*-silenced stem at 120 hour after attachment (haa): Appearance of stems that attach to *Nicotiana tabacum* expressing (A) empty vector and (C) *CcMCA1* amiRNA (Line si#2). Longitudinal sections of the stems that attach to *N. tabacum* expressing (B) empty vector control, and (D) *CcMCA1* amiRNA (Line #2, si#2). A photo in panel (D) is a composite of four serial pictures. Triangle: prehaustorium. In (C) and (D), the asterisk and the double asterisks indicate identical prehaustoria, respectively. In the stem of empty vector (A, C), prehaustoria developed in a uniform interval. In the *CcMCA1*-silenced stem (B, D), prehaustoria developed in varied intervals. In a gap between neighboring prehaustoria, no prehaustorial primordia was found in the central cylinder of the stem (dotted rectangle). This result indicates that haustorial development is repressed in its early stage in the *CcMCA1*-silenced stem, or alternatively, is retarded more than 120 hours.
