# Supplementary material for: Involvement of MID1-COMPLEMENTING ACTIVITY 1 encoding a mechanosensitive ion channel in prehaustorium development of the stem parasitic plant Cuscuta campestris
Source: Plant Cell Physiol. 2025 Jan 17;66(3):400–10. doi: 10.1093/pcp/pcaf009 (PMC11957263; doi:10.1093/pcp/pcaf009)
Supplement: pcaf009_Supp [file pcaf009_supp.zip › suppl_data/pcp-2024-e-00196-File013.pdf]

Park et al.
Supplementary Figure S5

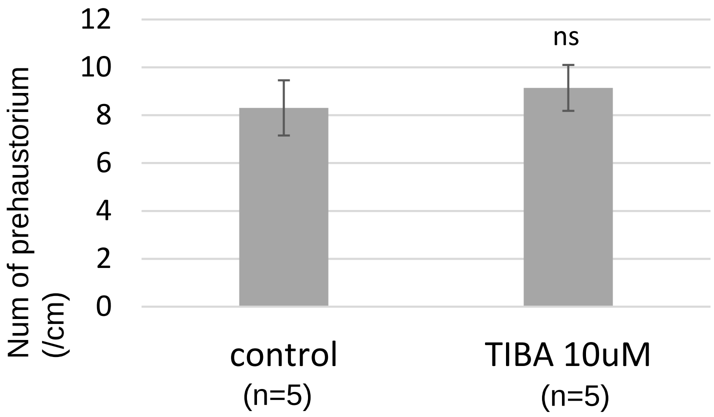

Supplementary Figure S5. Effect of 2,3,5-triiodobenzoic acid (TIBA) treatment on the prehaustorium density. Experimental setup was the same as shown in Figure 1. Parasitizing area were wrapped with filter paper soaked with either water or the inhibitor solution. Prehaustorium numbers per centimeter of the stem were counted at 48 hours after attachment (haa). ns: no significant difference at 5% level using Student's *t*-test.
