# Supplementary material for: Involvement of MID1-COMPLEMENTING ACTIVITY 1 encoding a mechanosensitive ion channel in prehaustorium development of the stem parasitic plant Cuscuta campestris
Source: Plant Cell Physiol. 2025 Jan 17;66(3):400–10. doi: 10.1093/pcp/pcaf009 (PMC11957263; doi:10.1093/pcp/pcaf009)
Supplement: pcaf009_Supp [file pcaf009_supp.zip › suppl_data/pcp-2024-e-00196-File014.pdf]

Park et al.

Supplementary Figure S6

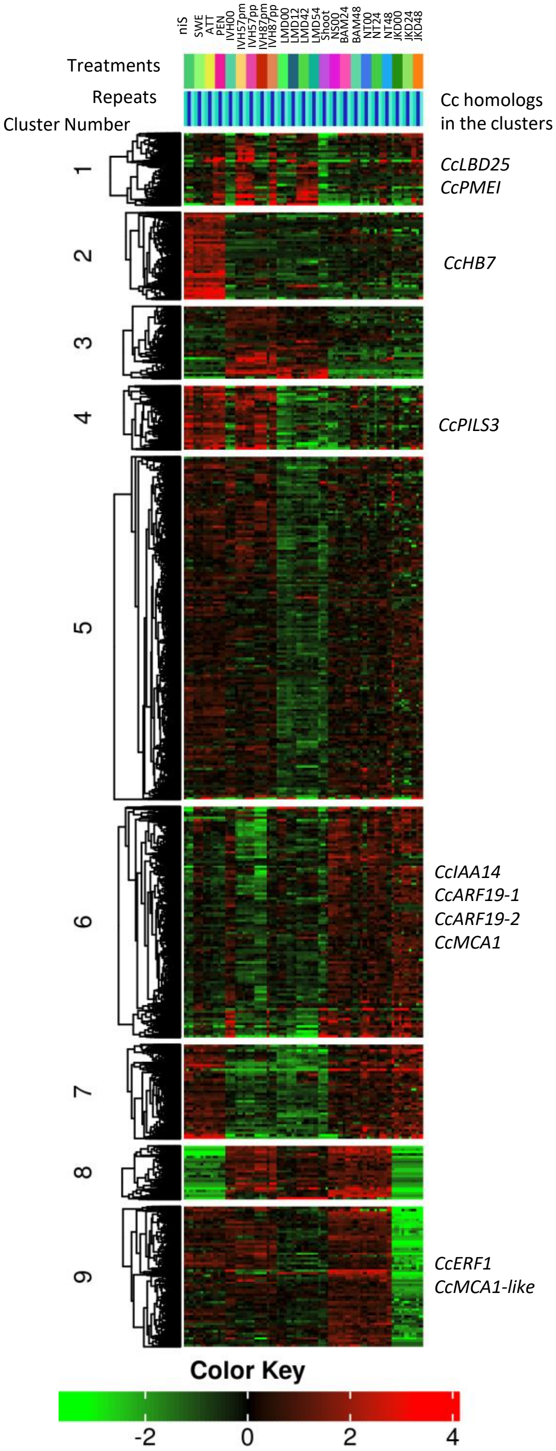

Supplementary Figure S6. K-means clustering of gene expression patterns in *Cuscuta campestris*. Gene expression data, provided in Supplementary Data S1, were combined with the mapping data from Bawin et al. (2022). For full descriptions of abbreviations, niS, SWE, ATT and PEN, see Bawin et al. (2022). The top 30,000 genes, ranked by standard deviations across samples, were used for clustering. Gene-by-gene correlation coefficients were calculated for genes in clusters 1, 6 and 9. Correlation coefficients for the gene to *CcLBD25* and *CcMCA1* are provided in Supplementary Data S2 and S3, respectively. Treatment: treatment of samples. Repeats: biological repeats of each treatment. All treatments consist of three repeats.
