# Supplementary material for: Involvement of MID1-COMPLEMENTING ACTIVITY 1 encoding a mechanosensitive ion channel in prehaustorium development of the stem parasitic plant Cuscuta campestris
Source: Plant Cell Physiol. 2025 Jan 17;66(3):400–10. doi: 10.1093/pcp/pcaf009 (PMC11957263; doi:10.1093/pcp/pcaf009)
Supplement: pcaf009_Supp [file pcaf009_supp.zip › suppl_data/pcp-2024-e-00196-File015.pdf]

Park et al.
Supplementary Figure S7

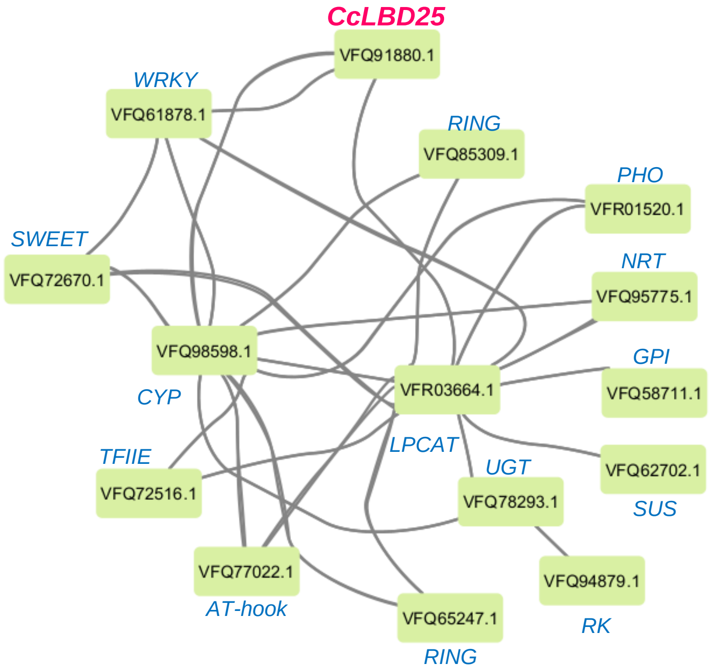

Supplementary Figure S7. Network of a co-expression module containing *CcLBD25*. Co-expression modules were generated using the Network tool in iDEP using parameters: Most variable genes to include = 3000, Soft Threshold = 5, Min. Module Size = 20. Co-expression module 21 (Supplementary Data S4), which includes *CcLBD25,* with an edge weight cutoff value of 0.24, was visualized using Cytoscape software. Node labeling (blue letters) indicate abbreviated gene names. *TFIIE*, Transcription initiation factor; *GPI*, GPI-anchored protein LLG1-like precursor; *AT-hook*, AT-hook motif nuclear-localized protein; *WRKY*, *WRKY* DNA-binding protein; *RING*, RING-H2 group; *UGT*, UDP-glucosyl transferase; *NRT*, nitrate transporter; *CYP*, cytochrome P450; *PHO*, phosphate transporter; *LPCAT*, lysophosphatidyl acyltransferase; *RK*, leucine-rich repeat protein kinase; *SWEET*, bidirectional sugar transporter *SWEET*; *SUS*, sucrose synthase;
